# An Essential Nuclear Protein in Trypanosomes Is a Component of mRNA Transcription/Export Pathway

**DOI:** 10.1371/journal.pone.0020730

**Published:** 2011-06-08

**Authors:** Mariana Serpeloni, Carolina Borsoi Moraes, João Renato Carvalho Muniz, Maria Cristina Machado Motta, Augusto Savio Peixoto Ramos, Rafael Luis Kessler, Alexandre Haruo Inoue, Wanderson Duarte daRocha, Sueli Fumie Yamada-Ogatta, Stenio Perdigão Fragoso, Samuel Goldenberg, Lucio H. Freitas-Junior, Andréa Rodrigues Ávila

**Affiliations:** 1 Departamento de Biologia Celular e Molecular, Universidade Federal do Paraná (UFPR), Curitiba, Brazil; 2 Laboratório de Regulação da Expressão gênica, Instituto Carlos Chagas (ICC), Curitiba, Brazil; 3 Center for Neglected Diseases Drug Discovery (CND3), Institut Pasteur Korea (IPK), Gyeonggi-do, South Korea; 4 University of Oxford, Oxford, United Kingdom; 5 Departamento de Biologia Celular e Parasitologia, Instituto de Biofísica Carlos Chagas Filho, Universidade Federal do Rio de Janeiro (UFRJ), Rio de Janeiro, Brazil; 6 Departamento de Bioquímica, Universidade Federal do Paraná (UFPR), Curitiba, Brazil; 7 Departamento de Microbiologia, Centro de Ciências Biológicas, Universidade Estadual de Londrina (UEL), Londrina, Brazil; State University of Campinas, Brazil

## Abstract

In eukaryotic cells, different RNA species are exported from the nucleus via specialized pathways. The mRNA export machinery is highly integrated with mRNA processing, and includes a different set of nuclear transport adaptors as well as other mRNA binding proteins, RNA helicases, and NPC-associated proteins. The protozoan parasite *Trypanosoma cruzi* is the causative agent of Chagas disease, a widespread and neglected human disease which is endemic to Latin America. Gene expression in *Trypanosoma* has unique characteristics, such as constitutive polycistronic transcription of protein-encoding genes and mRNA processing by trans-splicing. In general, post-transcriptional events are the major points for regulation of gene expression in these parasites. However, the export pathway of mRNA from the nucleus is poorly understood. The present study investigated the function of TcSub2, which is a highly conserved protein ortholog to Sub2/ UAP56, a component of the Transcription/Export (TREX) multiprotein complex connecting transcription with mRNA export in yeast/human. Similar to its orthologs, TcSub2 is a nuclear protein, localized in dispersed foci all over the nuclei —except the fibrillar center of nucleolus— and at the interface between dense and non-dense chromatin areas, proposing the association of TcSub2 with transcription/processing sites. These findings were analyzed further by BrUTP incorporation assays and confirmed that TcSub2 is physically associated with active RNA polymerase II (RNA pol II), but not RNA polymerase I (RNA pol I) or Spliced Leader (SL) transcription, demonstrating participation particularly in nuclear mRNA metabolism in *T. cruzi*. The double knockout of the TcSub2 gene is lethal in *T. cruzi*, suggesting it has an essential function. Alternatively, RNA interference assays were performed in *Trypanosoma brucei*. It allowed demonstrating that besides being an essential protein, its knockdown causes mRNA accumulation in the nucleus and decrease of translation levels, reinforcing that Trypanosoma-Sub2 (Tryp-Sub2) is a component of mRNA transcription/export pathway in trypanosomes.

## Introduction

In eukaryotic cells, transcription and translation are physically and temporally separated by the nuclear membrane. Control of mRNA transport to regions of translation defines when and where proteins are expressed. This transport is initiated in the nucleus, where association with protein complexes determinates the fate of mRNAs in the cytoplasm [1]. Different events in the nuclear metabolism of mRNA have crucial roles in controlling gene expression. The mRNA has to be correctly processed before being shuttled from the nucleus to the cytoplasm *via* a nuclear pore complex (NPC). The general model of RNA export involves exportins as transport receptors that carry RNA through the NPC in a RanGTP-dependent manner; specific exportins are involved with the different RNA types [2]. In contrast, nucleocytoplasmic export of most mRNAs does not follow the RanGTP-exportin pathway. In yeast and humans, mRNAs associate with protein factors as messenger ribonucleoprotein complexes (mRNPs) which are then exported through the NPC by an essential general receptor-shuttling heterodimer: Mex67/Mtr2 in yeast and TAP/p15 in humans [3], [4]. The dimeric export receptor operates in association with TREX (Transcription/Export), a multiprotein complex that connects transcription with mRNA export. Yeast TREX consists of the RNA helicase **Sub2** (UAP56 in human), the RNA-binding adaptor protein Yra1 (ALY in human), and the THO complex [5]–[7].

Excluding model eukaryotic organisms, the export machinery of other eukaryotes has yet to be determined. Using comparative genomics, we recently showed that the mRNA export pathway is the least conserved among early divergent eukaryotes, especially in excavates, a major kingdom of unicellular eukaryotes also known as Excavata [8]. In this lineage, we have suggested that mRNA export is quite different for several members. The phylogenetic category Excavata contains a variety of free-living and symbiotic forms, and also includes some major parasites affecting humans [9], [10].

The protozoan parasite *Trypanosoma cruzi* is a member of Excavata kingdom and the causative agent of the Chagas disease, a widespread and neglected disease in humans which is endemic to Latin America. Over 16 million people worldwide have been estimated to be infected with this parasite, and more than 100 million live in endemic areas [11]–[13]. *T. cruzi* gene expression displays some unique characteristics such as constitutive polycistronic transcription of protein-coding genes and mRNA processing by trans-splicing. Because genes in the same polycistronic unit display different levels of processed mRNA, the consensus notion is that post-transcriptional events are the major points of gene expression regulation and play important roles in the adaptation of the parasite throughout its life cycle [14], [15]. However, the machinery of the mRNA export pathway is not functionally studied in *T. cruzi*. We have previously shown that Sub2/UAP56, a component of the TREX complex, seems to be the most conserved protein among the factors involved in the mRNA export pathway [8]. In the present study we demonstrate, employing comparative modeling, that *T. cruzi* Sub2 (TcSub2) has a similar 3D structure when compared with the crystallized human ortholog. TcSub2 is a nuclear protein localized in foci dispersed all over the nucleus apart from the nucleolus. Furthermore, electron scanning microscopy revealed that TcSub2 is localized at the interface between dense and non-dense chromatin areas, a pattern of distribution typically associated with mRNA transcription/processing sites. BrUTP incorporation assays showed that TcSub2 colocalizes with RNA pol II transcription sites. These findings strongly suggest that TcSub2 is associated with transcription sites and may participate in nuclear mRNA metabolism. The double knockout of the TcSub2 gene is lethal in *T. cruzi*, indicating that, similarly to other eukaryotes, TcSub2 is an essential protein for trypanosomes. Alternatively, RNA interference (RNAi) assays in *T. bruceii* confirmed it has an essential function. Besides, its knockdown causes mRNA accumulation in the nucleus and decreasing of translation levels, confirming that this protein is a component of mRNA transcription/export pathway in trypanosomes.

## Materials and Methods

### *Trypanosoma* cultures

Epimastigotes of *T. cruzi* Dm28c [16] were cultured at 28°C in LIT medium supplemented with 10% bovine fetal serum [17]. The culture was seeded at 1×10^6^ cells ml^−1^ and the parasites were harvested when cultures reached a cell density of 1×10^7^ cells ml^−1^ (during log phase of growth). Different forms of parasite during metacyclogenesis were obtained *in vitro* as previously described [18], [19]. Amastigotes were obtained in LLC-MK2 (ATCC® – number: CCL-7™) [20] infected cells as previously described [21]. The culture of *T. brucei* procyclic forms of Lister 427 29-13 [22] were performed as previously described [23].

### Cloning and expression of TcSub2 recombinant protein for polyclonal antiserum production

The TcSub2 coding region (GenBank identification number 3538341) was amplified by PCR using the follow oligonucleotides: 5′GGGGACAAGTTTGTACAAAAAAGCAGGCTTCATGAGCAGTGGACTTGCCGAC3′ (forward) and 5′GGGGACCACTTTGTACAAGAAAGCTGGGTCCTATTACTGATTCATGTACTGGCTCTG3′ (reverse). Genomic DNA of Dm28c was used as a template. Both oligonucleotides contain recombination sites for cloning the TcSub2 ORF into the entrance pDONR vector (Invitrogen – Gateway® technology). This ORF was recombined into the expression vector pDEST™ 17, in accordance with the manufacturer's protocol for production of His_6_-tagged recombinant protein in the *Escherichia coli* BL21 (DE3) strain. The recombinant protein expression was induced with 1 mM IPTG for 3 h at 37°C; the protein was separated by SDS–PAGE, excised from the gel and eluted for inoculation in rabbit for polyclonal antiserum production.

### Immunoblotting assay

Cell lysates from 5×10^6^ parasites were loaded into SDS-PAGE and the proteins were transferred onto nitrocellulose membrane (Hybond C, Amersham Biosciences, England) using standard protocols. The membrane was incubated with the anti-TcSub2 polyclonal antiserum diluted 1∶1000 in blocking solution (5% of nonfat powdered milk, 0.1% Tween-20 in phosphate buffered saline, pH 7.4) for 1 h. After washing in PBS, the membrane was incubated with polyclonal anti-GAPDH antiserum diluted 1∶500 in blocking solution. This was then incubated for 45 min at room temperature (R.T.) with anti-rabbit horseradish peroxidase-conjugated IgG (Amershan Biosciences, England) diluted in PBS/Tween 0.5%. Detection of protein was performed according to the manufacturer's instructions.

### Comparative analysis by protein sequence alignments and molecular modeling

BLAST searches were carried out for TcSub2 *T. cruzi* protein— homologues at the GenBank databases (http://www.ncbi.nlm.nih.gov) using the sequences of the human and *Saccharomyces cerevisae* proteins as queries. Sequences were aligned using Clustal W (http://www.cmbi.kun.nl/bioinf/tools/clustalw.shtml) with homolog proteins of different organisms; including putative proteins of trypanosomatids and occasionally manual refinement of the alignments was performed. For the molecular modeling of TcSub2, the GenTHREADER software [24] was used to perform structural alignments based on the secondary structure of available proteins with known structure. The best match was the human HsaUAP56 (1XTK) protein [25]. The atomic coordinates from this structure were submitted with the alignment results to the program MODELLER [26] to produce the atomic-resolution models, which were then validated using the software PROCHECK [27], Verify 3D [28] and WHATIF [29].

### Ultrastructural immunocytochemistry

Parasites were fixed in 0.3% glutaraldehyde, 4% formaldehyde, and 1% picric acid diluted in 0.1 M cacodylate buffer (pH 7.2) and then dehydrated at −20°C in a graded series of ethanol solutions. The material was progressively infiltrated with Unicryl at lower temperatures and resin polymerization was carried out in BEEM capsules at 20°C for 5 days under UV light. Ultrathin sections were obtained with a Leica ultramicrotome (Reichert, Ultracuts) and grids containing the sections were incubated with 50 mM NH_4_Cl for 30 min. They were then incubated with blocking solution (3% BSA, 0.5% teleostean gelatin diluted in PBS, pH 8.0) for 30 min. This was followed by a 1 h incubation with anti-TcSub2 serum diluted 1∶200 in blocking solution. The grids were then incubated for 30 min with gold-labeled goat anti-rabbit IgG (Sigma) diluted 1∶200 in blocking solution. Grids were washed and stained with uranyl acetate and lead citrate for further observation in a Zeiss 900 transmission electron microscope. For control assays, incubation with the primary antiserum was omitted.

### *In situ* labeling of nascent RNAs

The assay was performed as previously described by Dossin and Schenkman (2005) [30]. The parasites were washed three times and resuspended to a cell density of 1×10^7^ ml^−1^ in transcription buffer containing 80 U µg ml^−1^ of RNasin (Promega). These cells were then permeabilized with 2.5 mg ml^−1^ of lysolecithin (Sigma) for 1 min on ice, centrifuged, washed in transcription buffer, and suspended to the same cell density in transcription buffer containing 2 mM ATP, 1 mM CTP, 1 mM GTP, 0.5 mM bromo-UTP (BrUTP) (Sigma), 200 mg ml^−1^ of creatine kinase (Roche), and 50 mM creatine phosphate (Roche). Cells were then incubated for 15 min at 28°C. As a control, the parasites were incubated in transcription buffer for 15 min with different concentrations of α–amanitin (Sigma) prior to the addition of nucleotides. Based on Campbell *et al.* (2003) [31], incubation of the parasites with 75 µg ml^−1^ of α–amanitin inhibits only RNA pol II while 200 µg.ml^−1^ inhibits both RNA pol II and III.

### Fluorescent microscopy analysis of TcSub2

For N-terminal fusion with GFP the TcSub2 ORF was cloned into the pTcGFPN vector [32] and the construct was transfected in *T. cruzi* epimastigotes as previously described [33]. For analysis of the cells expressing TcSub2-GFP, aliquots of cells were harvested, washed in PBS, resuspended in PBS to a density of 10^7^ cells ml^−1^, fixed with 4% paraformaldehyde in PBS for 20 min. The fixed cells were then placed onto poly-l-lysine-coated slides for 20 min at room temperature and were analyzed by fluorescence microscopy. For the indirect immunofluorescence (IF) assays, the cells were fixed as described above, permeabilized with 0.1% Triton X-100 in PBS for 5 min and blocked in 4% BSA in PBS for 60 min. For TcSub2 localization analysis, the cells were incubated for 45 min with anti-TcSub2 serum diluted 1∶1000. This was followed by a 45 min incubation with Alexa Fluor 488-conjugated donkey anti-rabbit IgG diluted 1∶1600 (Invitrogen) and 5 µg ml^−1^ DAPI. For transcription colocalization analysis, cells were incubated for 45 min with anti-TcSub2 serum diluted 1∶1000 and mouse anti-BrdU (Invitrogen) diluted 1∶300. This was followed by incubation for 45 min with Alexa Fluor 594-conjugated donkey anti-rabbit IgG diluted 1∶1600 (Invitrogen), Alexa Fluor 488-conjugated goat anti-mouse IgG diluted 1∶400 (Invitrogen), and 5 µg.ml^−1^ DAPI. All of the slides were mounted in VectaShield (Vector Laboratories). 2D images were collected with a Nikon E90i epifluorescence microscope using a 100×/1.4 PlanApoVC objective and a Nikon DS-QiMc camera. 3D images (z series) were collected with confocal Leica TCS SP2 AOBS using a 63x/HCX 1.4 PL Apo lbdBL oil immersion objective.

### TcSub2 knockout assay

Regions located upstream (UPS) and downstream (DWN) to the TcSub2 coding sequence were cloned into the pKS/NEO and pKS/HYG vectors which respectively contain the neomycin (NEO) and hygromycin (HYG) resistance genes [34]. The UPS and DWN were cloned in flanking regions of NEO or HYG genes. The oligonucleotides used for amplification of UPS and DWN are listed in the supplementary material (Table 1).

**Table 1:** Oligonucleotides used for amplification of UPS and DWN regions of the TcSub2 gene.

Oligonucleotides	Sequence (5′- 3′)	Enzyme	bp
**UPS-NEO F**	GGG**GTCGAC**GGTGTTATTTGCGTCACGATGTGC	***Sal*** **I**	**700**
**UPS-NEO R**	GGGC**GTCGAC**TTGCTTATGTCTATCTGGTTTCTTA	***Sal*** **I**	
**UPS HIGRO F**	GGG**TCTAGA**GGTGTTATTTGCGTCACGATGTGC	***Xba*** **I**	
**UPS HIGRO R**	GGGC**TCTAGA**TTGCTTATGTCTATCTGGTTTCTTA	***Xba*** **I**	
**DOWN-NEO F**	GGG**GAATTC**GATTTAAGGCCTCTGGAAAATAAGAATGAGG	***EcoR*** **I**	**450**
**DOWN-NEO R**	GGG**GAATTC**AACACAAAAATAAGAAGAAGTATTGGAATGGCAATTGC	***EcoR*** **I**	
**DOWN-HIGRO F**	GGG**GGATCC**GATTTAAGGCCTCTGGAAAATAAGAATGAGG	***Bam*** **HI**	
**DOWN-HIGRO R**	GGG**AAGCTT**AACACAAAAATAAGAAGAAGTATTGGAATGGCAATTGC	***Hind*** **III**	

NEO: neomycin; HIGRO: hygromycin. bp base pair. The enzyme cleavage sites are in bold.

The UPS/NEO/DWN construction (approximately 2100 bp) was PCR-amplified and purified by electroelution. A total of 10 µg of DNA was used to transfect *T. cruzi*, based on the method described by [33]. After 24 h, neomycin (G418 - Sigma, St. Louis, MO, USA) was added at a concentration of 300 µg ml^−1^ to select resistant parasites. The selected parasites were cloned by serial dilution in 24-well plates. Single allele knockout of TcSub2 was confirmed by Pulse-Field electrophoresis followed by Southern blot, where the TcSub2 and NEO genes were labeled with α-[P^32^]-dCTP (10 µCi/µl; 3000 Ci/mmol) and used as probes. Knockout was also confirmed by Western blot assays where protein extracts from knockout and wild-type parasites were used for detection of TcSub2, using GAPDH level as loading control. Comparative analysis for both proteins was performed through signal quantification using Scion Image program [35]. A single allele knockout parasites were then transfected with the UPS/HYG/DWN construction (approximately 2300 bp), which was PCR-amplified using the oligonucleotides UPSF e DWNR. The transfection was performed as described for the UPS/NEO/DWN construction, and 300 µg ml^−1^ G418 and 300 µg ml^−1^ hygromicin B (Sigma) were added to the LIT medium 24 h after transfection.

### RNAi assay in *T. brucei*

According to previous data of comparative genomics [8], *Trypanosoma brucei* has an ortholog protein (TbSub2) of TcSub2 with the accession number Tb10.70.7730 in GenBank database. For TbSub2 RNAi approach, the target sequence and primers were chosen using RNAit software [36]. The target sequence was PCR-amplified and cloned into the p2T7-177 vector [37]. The vector was linearized by *Not*I and transfected into procyclic forms of *T. brucei* 29-13 strain [22]. The stable DNA integration was selected using phleomycin (5 µg ml^−1^) and RNAi induction was performed after addition of 1 µg ml^−1^ of tetracycline. Cultures grown to mid-log phase (10^6^–10^7^ cells ml^−1^) were harvested for production of the total protein extracts. The knockdown of TbSub2 expression was confirmed by immunoblotting assay, using GAPDH level as loading control. Comparative analysis for both proteins was performed through signal quantification using Scion Image program [35].

### Fluorescent *in situ* hybridization (FISH)

For TcSub2- telomere colocalization analysis, log phase epimastigotes were subjected to indirect immunoflourescence followed by telomere detection using Telomere PNA FISH Kit/FITC (DakoCytomation) in accordance with the manufacturer's instructions. To observe colocalization of TcSub2 with SL transcription sites in *T. cruzi*, log phase epimastigotes were subjected to IF followed by Fluorescent *in situ* hybridization (FISH), using the SL-RNA as a probe. The SL-RNA probe was prepared by PCR using as template the *T. cruzi* (Y strain) SL RNA gene cloned in vector pGEM-T Easy. The SL probe was labeled with digoxigenin by PCR using High prime DNA labeling kit (Roche Diagnostics), purified and used in hybridization assays as described by Dossin et al., 2005 [30]. For detection of polyA-containing mRNA, FISH was performed based on previous works with modifications [38], [39]. Aliquots of cells (*T. cruzi* epimastigotes and *T. brucei* procyclic forms) were harvested, washed in PBS, resuspended in PBS to a density of 10^7^ cells ml^−1^ and fixed with 4% paraformaldehyde in PBS for 20 min. Fixed cells were washed three times in PBS, resuspended in 200 µl PBS and then placed onto poly-l-lysine-coated slides for 20 min at R.T. The cells were then permeabilized with 0.2% Triton X-100 in PBS for 30 min and washed three times with PBS. Pre-hybridization was performed in 10× Denhardt's solution, 4×SSC, 1 mM EDTA, 35% deionized formamide, 0.5 mg ml^−1^ tRNA, 2 mU ml^−1^ RNase OUT for 30 min at room temperature. For control, cells were previously treated with 100 µg ml^−1^ of boiled RNase A in PBS for 60 min at 37°C. Six ng/µl of digoxigenin (DIG) conjugated-5′ oligo d(T)_30_ was added to pre-hybridization buffer. The cells were heated to 65°C for 3 min before hybridization at R.T. overnight. The cells were washed twice with 2× SSC for15 min, twice with 1× SSC for 15 min, twice with PBS for 5 min and then incubated for 45 min at R.T. with anti-digoxigenin monoclonal antibody (Sigma) diluted in PBS/ BSA (0.1 mg ml^−1^). This was followed by incubation with Alexa Fluor 488-conjugated goat anti-mouse IgG diluted 1∶400 (Invitrogen) in PBS/BSA 0.1 mg ml^−1^ and 100 ng ml^−1^ DAPI for 45 min at R.T. The slides were mounted in N-propyl gallate and examined with a Nikon E600 microscope. Images were acquired with the Image Pro program (Media Cybernetics, Bethesda, MD, USA).

### Metabolic Labeling

The metabolic labeling of procyclic forms of *T. brucei* was performed according to [40] with modifications. A total 5×10^6^ cells from 48 h-induced and non- induced cultures was harvested from medium, washed in methionine-free RPMI medium (Sigma) supplemented with fetal bovine serum 0.5% and labeled by incubation, in the same medium containing 50 µCi ml^−1^ [^35^S]-methionine (Amershan Bioscience), for one hour at 28°C. For control, parasites were treated with 100 µg ml^−1^ of cycloheximide for 5 min before addition of [^35^S]-methionine. Aliquots of 10 µl were spotted in 3MM filters and boiled in 10% of trichloroaceticacid (TCA). After consecutives washes in 5% TCA, acetone an ethanol, the filters were dried at R.T. and the radioactivity was counted by liquid scintillation.

### Flow cytometry assays

All experiments were performed in a FACSCalibur flow cytometer (Becton-Dickinson, San-Jose, USA). A total of 20,000 events were acquired and data analysis was performed using FlowJo software (Treestar software). All experiments were done at least in duplicate.

Cell viability assays were performed using two parameters: propidium iodide staining and mitochondrial transmembrane potential [adapated from 41]. For the first assay, 2×10^6^ cells were harvested, washed with PBS and stained with 5 ng µl^−1^ propidium iodide in PBS at 28°C for 15 min. The cells were analyzed by flow cytometry using FL2-H detector. For the second assay, 2×10^6^ cells were harvested, washed in PBS and incubated with 10 µg ml^−1^ of rhodamine 123 in PBS for 15 min at 28°C. The cells were then resuspended in PBS for flow cytometry analyses using FL1-H detector. Data analysis was executed considering only viable cells gated by forward and side scatter. Relative mitochondrial membrane potential (in comparison to control cells) was determined considering the ratio (treated/control cells) of geometric mean of rhodamine 123 fluorescence.

The cell cycle analysis was performed by DNA staining with propidium iodide. 2×10^5^ cells were harvested, washed in PBS and resuspended in 500 µl of DNA staining solution containing 0.425 mM Tris-HCl, 9.375 µM Propidium Iodide, 0.0125% NP40 detergent, 1.25 mM NaCl and 8.75 U/L of Rnase A. The cells were kept on ice until immediate flow cytometry quantification. Data analysis was done in FL2-Widht×FL2-Area gated cells to exclude debris and doublets. The Dean-Jett-Fox algorithm of FlowJo software was used to estimate percentage of cells in G0/G1, S and G2/M phases of cell cycle.

Apoptosis was detected by phosphatidylserine exposure using BD PharMingenTM Annexin V-FITC Apoptosis Detection Kit according to manufacturer's instructions. Briefly, 2×10^6^ cells were harvested, washed and staining with annexin-V-FITC (AV) for 30 min at 28°C followed by staining with propidium iodide (PI) for 15 min at 28°C. Unstained and single-stained cells were used to set up compensation of signals from the detectors. To observe morphological alterations, cell samples from each assay were treated using the staining method “Panótico Rápido” (Laborclin, Pinhais, PR, Brazil) as described previously [34]. The stained parasites were examined in bright field using Leica SP5-AOBS confocal microscopy.

## Results

### Cloning of TcSub2 and comparative analysis of ortholog proteins by molecular modeling

**Figure 1:**
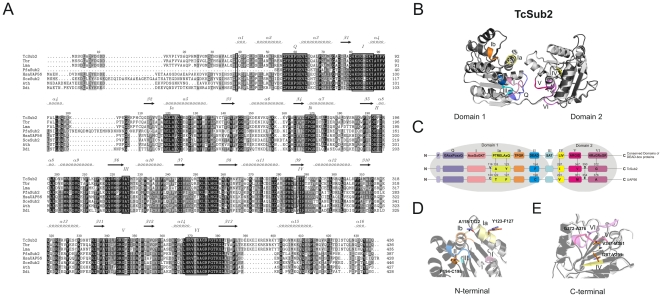
Comparison of amino acid sequences of TcSub2 with homolog proteins. (A) Amino acids are shaded according to their similarity: black for identical, dark grey if present in more than 60% of the sequences, and light grey if the similarity follows the BLOSUM 62 Matrix. When necessary, gaps were inserted within the various sequences (dashes) to allow alignment optimization. The nine motifs which are typical of DEAD-box RNA helicases are highlighted and predicted secondary structures are shown above the alignment. The GenBank accession numbers for the entries are listed as follows: TcSub2, (*T. cruzi* NP_808002.1); Tbr, (*T. brucei* XP_822312.1); Lma, (*Leishmania major* XP_001683110.1); PfaSub2, (*Plasmodium falciparum* XP_001349607.1); HsaUAP56, (Homo sapiens NP_004631.1); SceSub2, (*Saccharomyces cerevisiae* NP_010199.1); Ath, (*Arabidopsis thaliana* NP_568245.1); Ddi, (*Dictyostelium discoideum* XP_646415.1). (B) Molecular modeling of TcSub2 (light grey), based on the human homologue, HsaUAP56 (dark grey). Motifs are highlighted. Representations of the three-dimensional models were created with the software PyMol (http://www.pymol.org). (C) Analysis of TcSub2 motifs, compared with the general DEAD-box consensus and HsaUAP56 aminoacid disposition. (D–E). The arrangement of different amino acids that differ between TcSub2 (light grey) and HsaUAP56 (dark grey) are evidenced in N-terminal (D) and C-terminal (E) regions.

Previous studies have shown that only a small group of proteins involved in mRNA export is conserved throughout eukaryotic phylogeny. Among the components of the mRNA export pathway, Sub2 is the most conserved protein [8]. Its ortholog in *T. cruzi* (TcSub2) is annotated as a putative RNA helicase (Tc00.1047053508319.40) and is highly similar to SceSub2 (68%) and HsaUAP56 (69%). Multiple alignments of TcSub2 with similar proteins from different eukaryotes (Figure 1A) show a highly conserved aminoacid sequence through eukaryotic lineages. Sequence analysis revealed that TcSub2 exhibits nine canonical helicase motifs, typical of DEADX box-RNA helicase proteins, which are associated with nearly all processes involving RNA, from transcription to RNA degradation [42]. We obtained the molecular model of TcSub2 based on the crystal structure of human HsaUAP56 (Figure 1B). Comparative analysis between the molecular model of TcSub2 and HsaUAP56 shows a very conservative folding of RNA helicase motifs and two major helicase domains. Despite some amino acid differences between the sequences (Figure 1C) it does not seem to affect quality and overall aspect of the modeled structure of the TcSub2 RNA helicase (Figure 1D and E). Modifications in the N-terminal (regions Ia and II) are non-conservative (figure 1D) whereas all modifications of C-terminal regions (regions IV, V and VI) are conservative (figure 1E). In the N-terminal, the V116C substitution is located in a hydrophobic core and both A118T and Y123F are exposed to the solvent. These substitutions are therefore unlikely to cause any conformational changes to the structure as a whole. The substitution F194C, located in the region II, is part of a hydrophobic core and a few conservative substitutions surrounding the volumetrically bigger side chain of the phenylalanine is enough to accommodate this residue at that position. At C-terminal domain, the region V presents only one conservative substitution the V347M. This mutation is located next to one of the IPA biding sites of human structure but it is not directly related on its coordination. The substitution D350E is located between regions V and VI and it is structurally exposed, minimizing its impact on protein folding. The conservative variable residues for regions IV and VI (mutations I287V and G372A, respectively) are buried and could destabilize the TcSub2 structure. However, they are limited to the C-terminal domain and are therefore unlikely to cause disruption in the protein folding. Some of these aminoacid substitutions (Y123F, I287V, G372A) were also observed in the Sub2 ortholog of *Plasmodium falciparum* (pfU52) and did not affect the activity of the protein domains (Figures 1C–E). PfU52 also has RNA binding activity and is involved in ATPase and mRNA processing, similar to UAP56 in humans [43]. Since the TcSub2 structure is highly conserved, we assayed the functional ability of the TcSub2 protein to rescue the phenotype in *S. cerevisiae sub2* null mutants [44]. *SUB2* is an essential gene in yeast and the viability of the mutant cells is maintained by the episomal presence of the gene in a URA-3 plasmid. Functional complementation of heterologous genes in such strains can then be accessed by growth in FOA-containing plates. Since Sub2 levels are critical in yeast [45], we tested the expression of TcSub2 in low- and high-copy plasmids (as a control the *S. cerevisiae SUB2* gene was cloned into the same vectors). However, no growth was observed in any case even after 5 days of culture at 30°C (data not shown).

### TcSub2 is a nuclear protein localized in mRNA transcription sites

**Figure 2:**
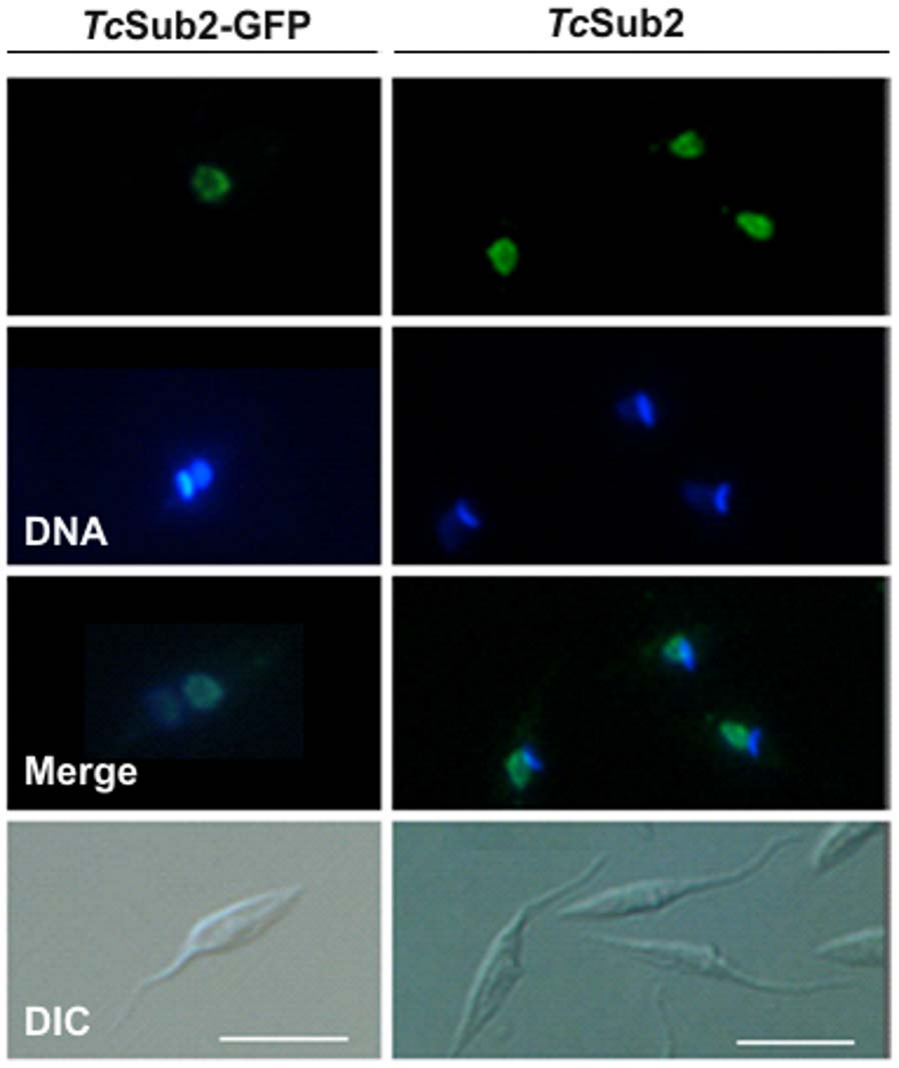
Localization of TcSub2 in *T. cruzi* Dm28c epimastigotes. The GFP fusion protein (TcSub2-GFP) and native protein (TcSub2) were localized by fluorescence microscopy. Specific polyclonal antibodies raised against TcSub2 were used to detect the native protein. Secondary antibodies were conjugated to Alexa-488. Cells were stained with DAPI to locate the nuclear and kinetoplast DNA. Bars = 10 µm.

**Figure 3:**
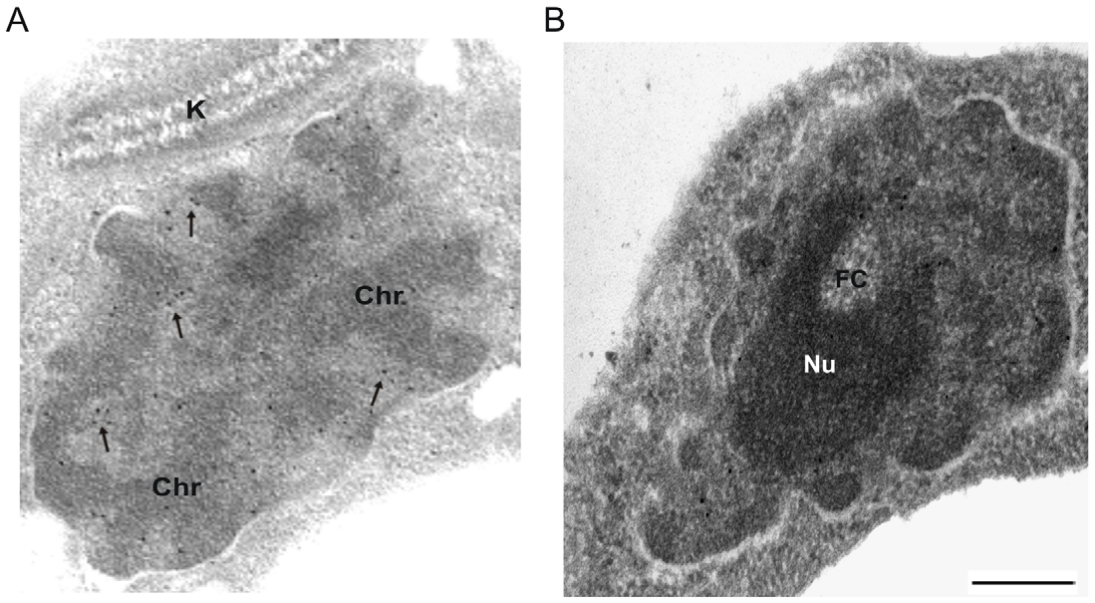
Immunoelectron microscopy localization of TcSub2 in *T. cruzi* Dm28c epimastigotes. (A) nuclear distribution of TcSub2: gold particles showing TcSub2 are seen at the edge of electron-dense chromatin (arrows). (B) Gold particles are present in the perinucleolar regions (arrows) and absent in the fibrillar center (FC) of nucleolus (Nu). Bars = 1 µm. k = kinetoplast, Chr = dense chromatin.

**Figure 4:**
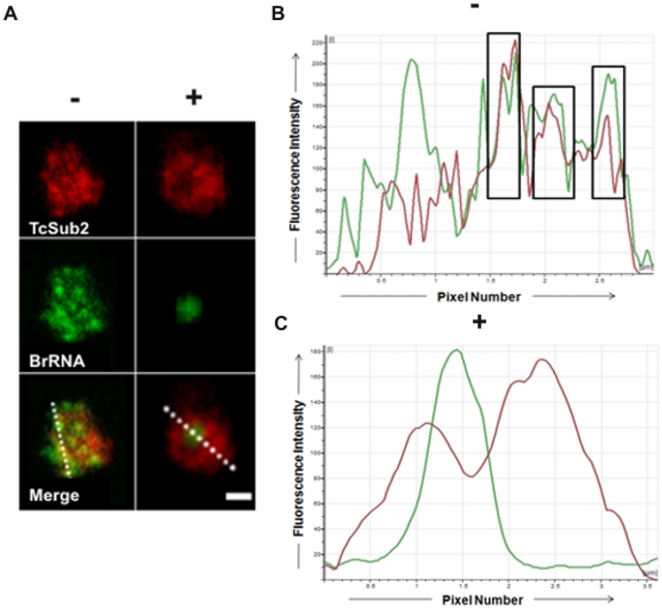
Nuclear colocalization of TcSub2 with active sites of transcription by detection of nascent RNAs in *T. cruzi*. (A) Immunolocalization of TcSub2 and active transcription sites (BrRNA) by confocal microscopy, without (−) and with (+) 200 µg ml^−1^ of α-amanitin. (B) Analysis of colocalization of TcSub2 (red curves) and transcription sites (green curves) without α-amanitin. Squares show colocalization of TcSub2 and transcription sites. (C) Analysis of colocalization of TcSub2 (red curves) and transcription sites (green curves) with α-amanitin. The fluorescence intensity profiles of TcSub2 and transcription sites show the distribution of fluorescence across the dotted line (x-axis). The fluorescence intensities are plotted along the y-axis and the squares indicate colocalization. Bars = 1 µm.

**Figure 5:**
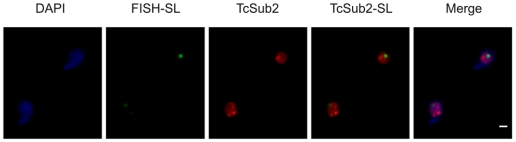
Nuclear colocalization of TcSub2 with SL transcription sites in *T. cruzi*. Immunofluorescence of TcSub2 (green) followed by FISH of SL transcription sites (red) was performed in epimastigote forms of *T. cruzi*. Cells were stained with DAPI to locate the nuclear and kinetoplast DNA. Bars = 1 µm.

The subcellular localization of TcSub2 was analyzed first by GFP fusion, where an exclusively nuclear distribution was observed by fluorescence microscopy in epimastigote forms (Figure 2, left). We observed that the protein is not evenly distributed in the nucleus, with some regions showing a more intense signal typical of a compartmentalized distribution. This distribution pattern was further confirmed by indirect immunofluorescence, using anti-TcSub2 polyclonal specific antibodies (Figure 2, right). This is a constitutive protein and the same pattern of protein localization occurs in different forms of the life cycle of the parasite and also in *Trypanosoma brucei* and *Leishmania major* (data not shown). To further investigate the nuclear distribution of TcSub2, ultrastructural immunocytochemical analyses were performed in *T. cruzi* epimastigotes (Figure 3). The results showed that this protein is localized mainly in less-compact chromatin regions (Figure 3A) and is absent from fibrillar center of the nucleolus (Figure 3B). Moreover, we observed the presence of grouped gold particles at the edge of electron-dense chromatin regions (Figure 3A). This distribution is similar to that of transcription sites [46], indicating that TcSub2 is located in active transcription regions. Analyses using indirect immunofluorescence combined with telomere FISH reinforced this notion because most of the protein does not colocalize with telomeres as indicated by the absence of TcSub2 signal over most of the telomeric repeats (Supplementary Figure S1). To test this hypothesis, *in situ* labeling of nascent RNAs followed by the immunofluorescence of TcSub2 was analyzed by confocal microscopy. This approach allowed the detection of BrUTP-labeled nascent RNAs (BrRNA), which corresponded to active transcription sites, and TcSub2 (Figure 4). The results showed that TcSub2 colocalizes with RNA pol II active transcription sites (Figure 4A). Based on previous work [31] incubation of the parasites with 75 µg ml^−1^ of α –amanitin inhibits only RNA pol II whereas 200 µg ml^−1^ inhibits both RNA pol II and III. We treated the cells using only 75 µg ml^−1^ of α –amanitin, but a very similar profile of signal detection was observed compared with the 200 µg ml^−1^ (data not shown). We therefore decided to use 200 µg ml^−1^ of the drug as the inhibition concentration for RNA pol II. When BrUTP incorporation was carried out in the presence of the RNA pol II inhibitor α-amanitin, there was no colocalization of TcSub2 to the RNA pol I transcription site. This confirmed our previous finding that TcSub2 is excluded from the fibrillar center of nucleolus. The distribution of TcSub2 in relation to RNA pol II and RNA pol I transcription sites is better observed by comparison of the fluorescence intensity profiles of confocal pictures of the BrUTP-labeled nuclei. In the absence of α-amanitin treatment, fluorescence intensity peaks of nascent RNAs and TcSub2 often correlated (Figure 4B). However, after α-amanitin treatment, poor correlation was observed between the BrRNA and TcSub2 fluorescent peaks (Figure 4C). This colocalization pattern was observed in different focal planes (Supplementary Figure S2). In addition, no colocalization between TcSub2 and Spliced Leader RNA was observed by FISH and immunolocalization analysis (Figure 5). Taking together, these results strongly suggest that TcSub2 is related to mRNA transcription and/or processing in the nucleus of *T. cruzi*. We also observed uncorrelated peaks of TcSub2 and BrRNA, suggesting that RNA pol II transcription sites are not the only nuclear domain associated with TcSub2.

### Trypanosome Sub2 ortholog proteins are essential for parasite survival

**Figure 6:**
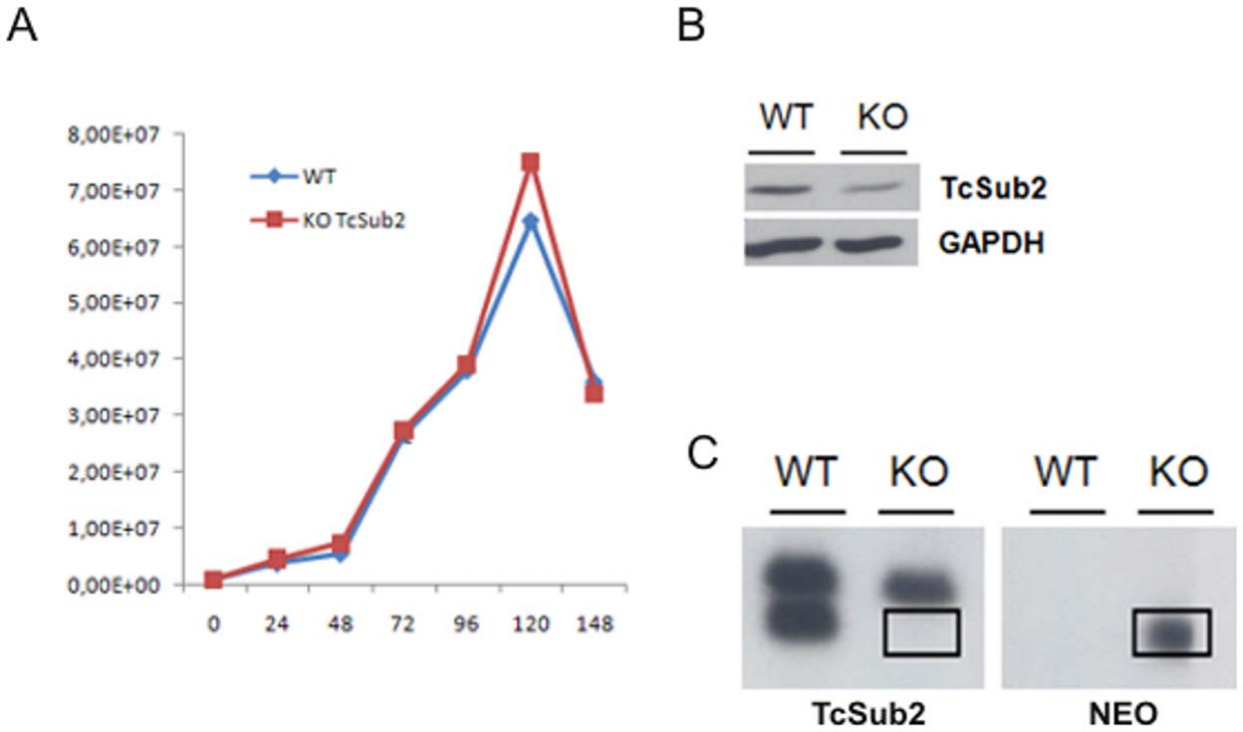
Cell proliferation analysis of single allele TcSub2 knockout of *T. cruzi*. (A) Growth curve of wild-type *T. cruzi* epimastigotes (blue squares) and single-allele knockout mutants (red squares). (B) Western-blot analysis comparing the expression of TcSub2 in wild-type (WT) and mutant parasites (KO). The membrane was incubated with 1∶1000 polyclonal anti-TcSub2 and anti-GAPDH serum. The level of TcSub2 protein was decreased in single-allele TcSub2-knockout parasites. (C) Southern blot analysis confirming the knockout of one allele of TcSub2. The membrane containing chromosomes of wild-type (WT) and single-allele knockout mutant was hybridized with TcSub2 (TcSub2) and Neomycin genes (NEO) labeled with ^32^P.

**Figure 7:**
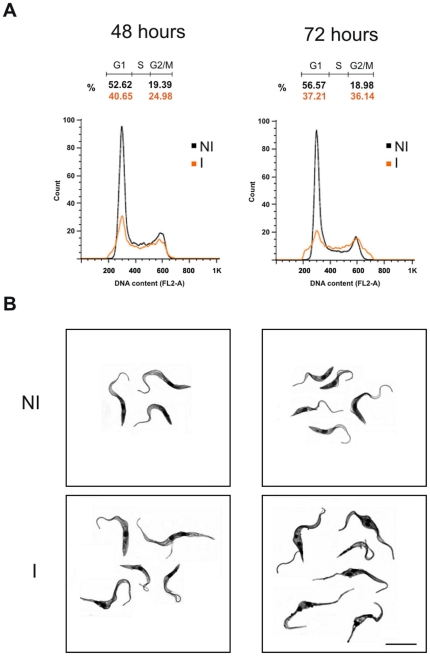
Analysis of cell cycle and morphology. (A) The cell cycle was analyzed by flow cytometry and the graphs correspond to data from RNAi assays after 48 and 72 hours of induction. Non-induced cells (NI) are indicated by black lines, induced cells (I) are indicated by orange lines. The cell cycle phases are indicated in each graph: G1, S and G2/M. (B) Morphology images from stained cells after 48 and 72 hours of RNAi induction obtained by confocal microscopy. Bars = 10 µm.

To analyze the function of TcSub2 we used a gene-knockout approach to abolish TcSub2 expression and observed the subsequent phenotypic alterations. A single-allele TcSub2 knockout of *T. cruzi* was successfully obtained (Figure 6). However, cell growth was not affected by the reduction in protein (Figure 6A). Western analysis using protein extracts from wild-type and single-allele knockout parasites, confirmed around 50% reduction on TcSub2 expression (Figure 6B). This reduction in protein expression is due to replacement of the TcSub2 allele by the neomycin selective marker as observed in the pulse field electrophoresis and Southern blot analysis (Figure 6C). We failed to achieve a null knockout because the cells were not viable during selection for hygromycin resistance. Three unsuccessful attempts were made to obtain the null knockout and we believe that this result is a consequence of the essential function of TcSub2 in *T. cruzi*. *Trypanosoma brucei* is a relevant model for analysis of gene function using an inducible system based on RNA interference [47]. Moreover, Sub2 is a highly conserved protein along eukaryotic evolution [8] and *T. brucei* has an ortholog protein which is 97% similar to TcSub2 (see alignment at Figure 1A). This encouraged us to use *T. brucei* as a suitable model to investigate the function of Sub2 in trypanosomes. First data showed that TbSub2 has an essential function since its knockdown in procyclic forms resulted in life cycle arrest in G2 phase (Figure 7A) with morphological changes (Figure 7B). Life cycle analysis by flow cytometry showed the presence of cells arrested in G2 phase (containing two nuclei and/or kinetoplasts) only after 48 h of RNAi induction, with an increasing of number of G2 arrested - cells after 72 h (Figure 7A). After 24 h of RNAi induction, the level of TbSub2 decreases around 45% and reaches 70% after 72 h of induction (Supplementary Figure S3A). The knockdown inhibits the cellular division after 24 h (Supplementary Figure S3B) but it does not trigger death by apoptosis (assessed by phosphatidylserine exposure, Figure S4). In addition, the analysis of cell viability by propidium iodide internalization (Supplementary Figure S3C and S5A) and mitochondrial transmembrane potential (Supplementary Figure S3D and S5B) shows that a strong increase in the percentage of non-viable cells occurs only after 96 hours of RNAi induction. Therefore, the results observed in the present study can not be explained by cell death patterns, since necrotic cell death should be a late secondary event due to knockdown of TbSub2.

### Knockdown of TbSub2 causes mRNA accumulation in the nuclei and translation inhibition

FISH assays to detect poliA^+^ RNA were performed to infer the distribution pattern of mRNA and investigate changes after decreasing Tryp-Sub2 protein levels in the parasite. As shown in supplementary figure S6, the distribution patterns of mRNA are very similar between wild type and single knockout cells of *T. cruzi*. It is observed a major signal in the cytoplasm and around the nucleus, but a very weak signal inside the nucleus. The analysis was done at different time-points of growth but no evident changes were observed.

**Figure 8:**
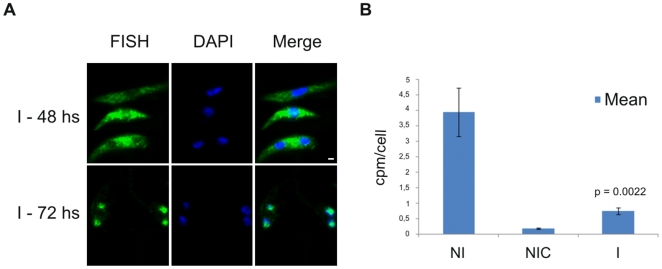
FISH of poliA^+^mRNA and translation level after TbSub2 knockdown. (A) Fluorescence microscopy of poliA^+^ mRNAs by FISH using DIG-labeled oligo d(T)_30_ after 48 and 72 hours of RNAi induction. Cells were stained with DAPI to localize the nuclear and kinetoplast DNA. Bars = 1 µm. (B) Metabolic labeling after 48 hours of RNAi induction. The graph shows the average of radioactive counting from triplicate experiments of non-induced cells (NI), non-induced cells treated with 100 µg ml^−1^ of cycloheximide (NIC), and induced cells (I). The p value, calculated by unpaired t-test, is indicated in the graph.

By contrast, the knockdown of TbSub2 affected the mRNA distribution pattern in *T. brucei*. Differently from wild type cells, after 48 h of RNAi induction it is possible to observe a slight signal increase inside the nucleus (Figure 8A, on the top; Supplementary Figure S7); however the signal is still visible in the cytoplasm. This might correspond to mRNA accumulation in the nucleus that is clearly visible after 72 h of induction. (Figure 8A, below; Supplementary Figure S7). By analysis using metabolic labeling with [^35^S]-methionine, it was demonstrated that the silencing of TbSub2 expression also caused translation inhibition after 48 h of induction (Figure 8B). It was possible to observe a deep decrease of radioactive counting in induced cells when compared to non-induced cells, similar to the result observed for non-induced cells treated with the translation inhibitor cycloheximide. We believe that the translation inhibition and mRNA accumulation is not due to cell death since knockdown of TbSub2 strongly affects the cell viability only after 96 h of induction, as shown before (Supplementary Figure S3C,D; Supplementary Figures S4 and S5).

## Discussion

Post-transcriptional events are crucial for regulation of gene expression in trypanosomatids because of the absence of specific control mechanisms during transcription. Unlike most eukaryotic organisms, in which each gene transcribed by RNA Pol II has its own promoter, transcription in trypanosomatids is polycistronic without traditional promoter elements and genes in individual clusters do not necessarily code for functionally related proteins [15]. Mature mRNAs are generated from primary transcripts by trans-splicing and polyadenylation and are then moved to the cytoplasm to be translated [48]. In the context of post-transcriptional events, the machinery of mRNA export is poorly understood in trypanosomes and this pathway might be an important step in regulation of gene expression in these parasites. We were therefore interested in investigating the factors that could be involved in this pathway.

The export of a few mRNAs in *T. cruzi* can be mediated by CRM1, a component of the RanGTP-exportin pathway. This pathway is commonly responsible for protein export but has no major role in mRNA export in higher eukaryotes [49]. Most reports related to this topic come from model organisms, especially *S. cerevisiae*, and the bulk of mRNA is exported in a RanGTP independent pathway involving the THO/TREX complex [revised by 2]. Based on our previous investigations, we started biological analysis of the most conserved component of the eukaryotic mRNA export pathway, the yeast DEAD-box RNA helicase Sub2 (UAP56 in humans). In the present study we cloned the gene encoding the *T. cruzi* protein that is highly similar to Sub2/UAP56, and has been named TcSub2. Sub2/UAP56 is a component of the TREX multiprotein complex that links transcription with mRNA export [revised by 50]. DEAD-box proteins are involved in the ATP-dependent unwinding of double-stranded RNA (dsRNA), displacement, RNA remodeling, or RNA/protein complexes. They are characterized by nine conserved motifs distributed in two domains. In general, motifs I and II are implicated in ATP binding and hydrolysis, with contributions from motif VI. Motif III is believed to couple ATP hydrolysis with RNA unwinding, whereas motifs IV, V, and VI contribute to RNA binding [42], [51]. We noted some changes of amino acid composition in the TcSub2 sequence when compared to the human sequence (UAP56). However, these changes did not affect the molecular model of TcSub2 that is very similar to the UAP56 crystal structure.

Although TcSub2 is highly conserved, it is unable to function as a substitute for SUB2. We speculate that this could be due to the specific functions of TcSub2 in *T. cruzi* because most transcripts in this parasite are processed by *trans*-splicing rather than *cis*-splicing as in other eukaryotic cells [52]. Specific point mutations could be enough to create specific characteristics in the TcSub2 protein, and could, for example, prevent the interaction of this protein with components of TREX from *S.cerevisiae*. Such an example has been recently reported by Vázquez and colleagues (2009) [53]. TcU_2_AF35, which is involved in the initial steps of *trans*-splicing in *T. cruzi*, is able to functionally complement yeast cells when only two aminoacid residues are modified. This demonstrates the importance of conserved residues for functional substitution of ortholog proteins between different organisms. Another possibility might be the development of novel components in the *T. cruzi* pathway, where specific factors would be necessary for functional activity of TcSub2. In the export of mRNA, there is one revealing example involving the yeast Mex67-Mtr2 protein, an adaptor which is responsible for mRNA export, and its human counterpart, TAP-p15. TAP alone is unable to complement mex67- mutant cells neither does p15 complement mrt2^−^ mutants. However, the TAP-p15 complex partially restores the growth defect of mex67^−^/mtr2^−^ double mutants showing that TAP and p15 must interact to be functional in yeast whereas the TAP-Mtr2 and Mex67-p15 complexes may not form [54]. Like Sub2 and UAP56, TcSub2 is exclusively nuclear and is dispersed in loci all over the nuclei, and it is also present at the periphery of nucleolus, excluding the fibrillar center of nucleolus.

The ultrastructural immunocytochemical assays showed that TcSub2 is concentrated in non-dense chromatin areas and grouped mainly at the interface between dense and non-dense chromatin. This speckled pattern of distribution is usually associated with mRNA transcription, processing, and nucleocytoplasmatic export [45], [55], [56]. This distribution pattern is similar to human and arthropod (C*hironomus tentans*) TcSub2 homologues which are localized on the periphery of dense chromatin domains, termed interchromatin granule clusters (IGCs) [57]. These clusters contain mainly proteins related to mRNA processing, especially SC-35, and are closely related with perichromatic fibrils, where mRNA transcription occurs [revised by 58], [59]. Many studies have demonstrated that nascent mRNAs are deposited in these interchromatin spaces [46], [60]–[62]. A similar distribution pattern has been observed in trypanosomatids, at the interface between dense and non-dense chromatin for bromodomain factors (BDFs) and acetylated histones [63]–[65]. Recent investigations show that these histone modifications can serve as indicators of regions for initiation of RNA Pol II transcription [64], [66], [67]. Based on our findings for TcSub2 localization, we decided to investigate the relationship of TcSub2 and active transcription sites using BrUTP incorporation followed by immunocolocalization. This approach has been successfully used to observe the association of RNA Pol II transcription sites with proteins such as Hrp59 and Hrp65, and hnRNPs in *C. tentans*. It has also been used to define the localization of RNA Pol II transcription sites in *T. cruzi* [30], [68], [69]. Our results demonstrated that TcSub2 also colocalizes with nascent RNAs —specifically with those transcribed by RNA pol II— responsible for mRNA, snRNA and spliced-leader (SLRNA) transcription. Blocking of RNA pol II activity with α-amanitin abolished the transcription of nascent RNAs, resulting in RNAs transcribed only by RNA pol I. Under these conditions, we observed the absence of colocalization with TcSub2, indicating that TcSub2 might be associated with RNA pol II, but not with RNA pol I transcription. Besides, the protein does not localize with SL-RNA, reinforcing that the protein is related to mRNA transcription sites. The association of TcSub2 with RNA pol II transcription sites strongly suggests that TcSub2 is functionally similar to homolog proteins in other eukaryotes. However we are still unable to confirm where TcSub2 is involved in the pathway of nuclear mRNA metabolism in this parasite. We also observed uncorrelated peaks of TcSub2 and BrRNA, suggesting that RNA pol II transcription sites are not the only nuclear domain associated with TcSub2. We speculate that the partial association with nascent mRNAs can be explained by the dynamics of interaction between different events in the cell, where DNA regions could be at different stages of transcription. Another explanation could be that TcSub2 is related to transcription of specific mRNAs as consequence of its role in regulation of gene expression. To further investigate the function of TcSub2, we attempted to knockout the TcSub2 gene to obtain phenotypic alterations related to mRNA export. However, the null knockout lineages of *T. cruzi* were not viable. Studies show that it is an essential protein in other eukaryotes because the knockdown of Sub2 homologs inhibits cell growth [45], [55], [70], [71]. Unfortunately, knockout of a single TcSub2 allele was not enough to affect morphology and cell growth. Although the single-allele TcSub2 knockouts had around 50% less TcSub2 protein, this did not alter mRNA export. It is therefore necessary to apply a regulated system to completely silence TcSub2 expression to get more information regarding to its function in *T. cruzi*. The absence of approaches of inducible silencing, such as RNA interference, in *T. cruzi* preclude suitable studies for this. On the other hand, *T. bruceii* is a suitable model to study essential genes using inducible system based on RNAi in trypanosomes. Taking in account that Sub2 is highly conserved in trypanosomes [8], we decided to use *T. bruceii* to study the function of this protein. The data obtained demonstrated that it is really an essential protein since its knockdown inhibits the cellular growth. Similar to other eukaryotes, the silencing of TbSub2 expression caused translation inhibition and robust accumulation of bulk polyadenylated mRNAs in the nucleoplasm. These phenotypes are also observed when the expression of homologs is silenced in metazoan [45], [55], [71]–[76]. Interestingly, we also observed an arrest of cell cycle in G2 phase, mainly after 72 hours of RNAi induction when the level of the proteins was reduced by 70%. In the same way, knockdown of UAP56, the homolog in humans, resulted in mitotic defects. Taking together, the trypanosome protein has similar characteristics to its orthologs in higher eukaryotes, possibly reflecting a similar function in these parasites.

In the context of nuclear mRNA metabolism in trypanosomatids, nucleocytoplasmic transport is not well understood and data in this area will lend further insights into regulation of gene expression in these parasites. Herein, we identified a novel trypanosome protein that it is related to nuclear mRNA metabolism and proposed that the Tryp-Sub2 is a component of mRNA transcription/export pathway in these parasites.

## Supporting Information

Figure S1 **Nuclear colocalization of TcSub2 with telomere repeats.** FISH of telomeric repeat regions followed by immunofluorescence of TcSub2 was perfomed in epimastigote forms. Telomeric repeats (red); TcSub2 (green). Cells were stained with DAPI to locate the nuclear and kinetoplast DNA. Bars = 1 µm.
(TIF)

Figure S2 (A, B) **Nuclear** c**olocalization of TcSub2 with transcription sites in different focal planes.** Analysis of colocalization of TcSub2 (red) with transcription sites (BrRNA, green) without α-amanitin. Bars = 1 µm.
(TIF)

Figure S3 **Cell proliferation and viability analysis.** (A) Growth curves of induced (I) and non-induced (NI) cells by direct counting in a Neubauer chamber; each point represents the mean and standard deviations of triplicate experiments. Western blot of TbSub2 during RNAi induction. Antiserum against GAPDH was used as a protein-loading control. (B) Percentage of cells in G2 phase of cell cycle calculated by flow cytometry (details in methods); (C) Percentage of non-viable cells (dead cells) calculated by flow cytometry using propidium iodide staining; (D) Comparison of R123 fluorescence intensity between induced to non induced cells plotted as fold change graph using geometric mean of FL1-H detector intensity. For flow cytometry data (B–D), each experimental point represents the average and respective standard deviation of duplicate experiments.
(TIF)

Figure S4 **Analysis of phosphatidylserine exposure after TbSub2 knockdown by RNAi.** The density plots show the non-induced (NI, top row) or induced (I, bottom row) cells co-stained with Annexin-V-FITC and Propidium Iodide after 48 to 96 hours (indicated above the plots) of RNAi induction. The values inside the density plots represent the percentage of cells inside each region.
(TIF)

Figure S5 **Flow cytometry analysis of cell viability and cell cycle after TbSub2 knockdown by RNAi.** (A) Overlay histograms of induced (I) and non-induced (NI) cells stained with Propidium Iodide after 24 to 96 hours (h, indicated inside the graphs) of RNAi induction; the percentage of PI positive cells (“Dead” region) is indicated. (B) Overlay histograms of I and NI cells stained with rhodamine 123 for mitochondrial membrane potential analysis. (C) Analysis after RNAi induction of DNA content by staining of permeabilized cells with propidium iodide; note the gradual increase of cells in G2/S phase after 48 hours of RNAi induction. (D) Cell cycle analysis by Dean-Jett-Fox algorithm of FlowJo software; data of non-induced (left histogram) and induced (right histogram) cells after 48 hours of RNAi induction are shown; the percentage of cells in each phase of cell cycle is indicated inside the graphs.
(TIF)

Figure S6 **FISH of poliA^+^mRNA in** ***T. cruzi*** **.** Localization of mRNAs by FISH using DIG-labeled oligo d(T)_30_ in wild-type (WT) and single knockout of TcSub2 (KO) epimastigotes. Cells were stained with DAPI to locate the nuclear and kinetoplast DNA. For control, cells were treated with 100 µg ml^−1 of^ RNAse A before hybridization. Bars = 1 µm.
(TIF)

Figure S7 **FISH of poliA^+^mRNA in** ***T. brucei*** **.** Localization of mRNAs by FISH using DIG-labeled oligo d(T)_30_ after 48 and 72 hours of RNAi induction. Cells were stained with DAPI to locate the nuclear and kinetoplast in non-induced cells (NI) and induced cells (I). DNA. For control, cells were treated with 100 µg ml^−1^ RNAse A before hybridization. Bars = 1 µm.
(TIF)
